# Quality of life and healthcare utilization during the COVID-19 pandemic are more restricted in chronically ill than in healthy children: a tertiary care children’s hospital experience

**DOI:** 10.1007/s00431-023-05382-6

**Published:** 2024-01-23

**Authors:** Johannes Hilberath, Anna-Sophia Mast, Maximilian Holweg, Lara Kränkel, Jonathan Remppis, Hanna Renk, Peter Lang, Johannes Schulte, Jörg Fuchs, Christoph Slavetinsky

**Affiliations:** 1 Department of Hematology and Oncology, University Children’s Hospital Tübingen, Hoppe-Seyler-Straße 1, 72076 Tübingen, Germany; 2 Pediatric Surgery and Urology, University Children’s Hospital Tübingen, University of Tübingen, Hoppe-Seyler-Straße 3, 72076 Tübingen, Germany; 3 Institute of Medical Microbiology and Hygiene, University Hospital Tübingen, Tübingen, Germany; 4 University Children’s Hospital Tübingen, Hoppe-Seyler-Straße 1, 72076 Tübingen, Germany

**Keywords:** COVID-19, Chronically ill children, Quality of life, Healthcare utilization

## Abstract

The global COVID-19 pandemic forced changes in everyday life of children and adolescents due to government containment measures, an altered healthcare accessibility and utilization, and public concern about SARS-CoV-2 transmission. Data on the challenges and impact on children and their families with chronic diseases are limited. The primary objectives of this study were to assess (i) concerns for SARS-CoV-2 infection, (ii) perceived effects on health-related and overall quality of life (HRQoL and QoL), and (iii) accessibility and utilization of healthcare, comparing families with chronically ill children to families with healthy children during the second SARS-CoV-2 infection wave in Germany. A caregiver questionnaire was designed and participation offered in the emergency department and outpatient clinic of a German tertiary care children’s hospital. 45.9% of the 205 participants were majorly concerned about their children contracting a SARS-CoV-2 infection. Caregivers of chronically ill children (128/205, 62.4%) stated significantly more often a negative impact on their child’s QoL (*w* = 0.17; *p* = 0.014), while caregivers of chronically ill adolescents over the age of 13 expressed significantly more frequent a negative impact on their child’s HRQoL (*w* = 0.21; *p* = 0.016). Outpatient appointments for chronically ill children were significantly more often canceled (*w* = 0.17; *p* = 0.025). Caregivers of chronically ill children were significantly more likely to report that they would actively delay hospital visits for emerging health issues due to the pandemic (*w* = 0.12; *p* = 0.049).

*     Conclusion*: Our findings underscore the importance of identifying families with chronically ill children as a vulnerable patient group with higher burdens during the COVID-19 pandemic and potential future pandemics. Healthcare providers may mitigate such burdens by ensuring reliable appointment allocation, offering contactless healthcare options, and providing tailored advice regarding vulnerabilities and preventive measures specific to their chronically ill children.

**Table:** 

**What is Known:** *• The SARS-CoV-2 pandemic has led to significant restrictions in everyday life and both accessibility and utilization of healthcare for children and adolescents.* *• Chronically ill children faced exceptional challenges as they depend on regular and functioning medical care, but data comparing the pandemic’s impact between chronically ill and healthy children are lacking.*	
**What is New:** *• The perceived impact of the SARS-CoV-2 pandemic on quality of life is more negative for chronically ill children and their health-related quality of life is more often affected compared to healthy children.* *• Caregivers of chronically ill children would more often delay a visit to their child’s doctor during the SARS-CoV-2 pandemic and their medical appointments are more often postponed which both could increase health burdens for such vulnerable patients.*

**Supplementary Information:**

The online version contains supplementary material available at 10.1007/s00431-023-05382-6.

## Background

The coronavirus disease 2019 (COVID-19) pandemic caused by the severe acute respiratory syndrome coronavirus 2 (SARS-CoV-2) and the associated containment measures to prevent the spread of the virus profoundly influenced daily routine of individuals and families worldwide [1].

Children of all ages are at risk for COVID-19 [2], although the disease course is often milder and the prognosis better than in adults [3]. However, severe medical courses do occur, especially in high-risk patient groups including children with underlying diseases [4, 5]. In addition, serious secondary phenomena following COVID-19 such as the pediatric inflammatory multisystem syndrome temporally associated with SARS-CoV-2 (PIMS-TS) and long COVID have been documented in children [6–8]. While the number of emergency department visits markedly dropped during the pandemic, there is evidence that caregivers delay presentation of their children to the emergency departments due to fear of contracting SARS-CoV-2 [9, 10]. Furthermore, as part of the containment measures, access to specialized medical care was restricted [11]. Reduced access to healthcare services is especially demanding for children with chronic diseases that require regular appointments and high standard of medical care.

Children and adolescents experienced a changed psychosocial environment and high levels of socio-emotional stress due to social distancing, lockdown and containment, isolation, temporary school and kindergarten closures as well as restrictions in activities [1, 12, 13]. Two nationwide representative studies of German and Norwegian children and adolescents reported high burdens caused by the pandemic and additionally led to significantly lower health-related quality of life than prior to the pandemic [14, 15].

Evidence on the impact of the COVID-19 pandemic on families with chronically ill children is limited [16, 17]. To our knowledge, there is no study evaluating and comparing the perceived impact on quality of life and healthcare utilization of their children among both, parents caring for chronically ill children and parents with healthy children.

The primary objectives of this study were to assess the differences between participants with chronically ill compared to participants with healthy children during the COVID-19 pandemic analyzing (i) concerns of contracting a SARS-CoV-2 infection, (ii) caregivers-reported health related and overall quality of life (HRQoL and QoL) of their children, and (iii) utilization and accessibility of medical care. The study was conducted spanning the second SARS-CoV-2 infection wave in Germany from October 2020 to March 2021 when infection numbers starting increasing again for the third wave [18–20]. While during the second wave, no vaccination was available for the broad population, there was a substantial rise in COVID-19-related deaths, a massive impairment of mental health in the German population, and ongoing COVID-19-related restrictions in daily life [18].

## Methods

### Study design and questionnaire

The present study reports data from a large tertiary care facility center with a total of 170 beds, treating more than 20.000 outpatients annually (University Children’s Hospital, Tübingen, Germany). Data was collected prospectively from October 29, 2020 to March 23, 2021, starting 7 months after beginning of the first infection wave of COVID-19 and spanning over the second infection wave in Germany. With support of the Institute of Occupational and Social Medicine and Health Services Research and the local Centre for Pediatric Clinical Studies at our hospital, we designed a paper-and-pencil questionnaire to assess the caregivers perceived impact of the COVID-19 pandemic and the associated restrictions on HRQoL and overall QoL among their chronically ill and healthy children. The questionnaire was validated concerning its content, internal consistency, and construct validity (Supplementary file 1). The study was conducted according to the ethical principles of the Declaration of Helsinki and approved by the independent ethics committee of the Medical Faculty of the University of Tübingen (Project Number 686/2020BO2).

Anonymous participation in the questionnaire was offered by convenience sampling to caregivers of patients presenting in the emergency department or outpatient clinics. Questionnaires were distributed in a blinded fashion, without knowledge whether caregivers presented with healthy or chronically ill children. Exclusion criteria were the inability to speak and read German not meeting at least reference level B2 defined by the Common European Framework of Reference for Languages (CEFR), not being the child’s legal caregiver, and an incorrectly completed questionnaire. Response options were designed in a multiple-choice format containing precise options (Supplementary files 1 & 2). For questions in which a grading system was required, a 5-level Likert scale was implemented. Likert items were designed as bipolar scaling system ranging from high agreement to high rejection either ranging from “very high,” “high,” “moderate,” “low,” to “not at all” when assessing the infection risk, or from “very much,” “much,” “moderate,” “low,” to “not at all” when assessing the impact on (HR)QoL. The answer item “I do not know/no information” was offered in all (appropriate) questions, including “yes” or “no” questions. One answer only was possible in most questions. Multiple answers were possible in 2 questions, which was explicitly mentioned before the respective question. Some questions contained specific linear answers. The first part of the questionnaire comprised questions about the participants self-reported sociodemographic parameters and whether they were taking care of a child with a chronic disease, being defined as a condition lasting longer than 3 months and restricting age-appropriate activities and/or requiring regular health checks [21]. The second part contained questions about the caregivers’ concerns of themselves or their children contracting COVID-19 as well as the perceived impact of the COVID-19 pandemic and the associated restrictions on HRQoL and QoL of their children (proxy-reported (HR)QoL). Lastly, caregivers answered questions concerning the postponement of healthcare appointments, their utilization of medical care during the COVID-19 pandemic, and their demand for more contactless healthcare options.

### Statistical analyses

Caregivers’ sociodemographic variables were evaluated by descriptive analysis stratified whether the caregivers reported caring for chronically ill or healthy children and presented as counts and percentages. Associations between variable pairs were evaluated using a chi-square test for categorical variables (Pearson’s chi-squared test, degrees of freedom (df) = 1) and Mann–Whitney *U* test for analysis on Likert scale type questions. A *p* value < 0.05 was defined as statistically significant. Microsoft Excel (Microsoft Excel 2010 v14.0, Microsoft Corporation, Redmond, WA, USA), SPSS (IBM SPSS Statistics 26, IBM, Armonk, NY, USA), and GraphPad Prism (GraphPad Prism version 9.4.1, GraphPad Software, Boston, MA, USA) were used for data collection and analysis.

## Results

### Participant characteristics

Between October 2020 and March 2021, spanning the second SARS-CoV-2 infection wave in Germany, 229 caregivers presenting with their child to our emergency department or outpatient clinic were invited to answer our questionnaire. Five persons declined to participate, and 19 questionnaires were filled out incorrectly (defined as incomplete or unclearly filled out questionnaire, or checking multiple answers if just one answer was appropriate), resulting in exclusion from the analysis. Therefore, 205 questionnaires were evaluated, containing the answers of 128 caregivers with chronically ill and 77 caregivers with healthy children (Table 1). The answer option “I do not know/no information” was rarely given by the participants (on mean 3.5% of all answers per question). Among caregivers with chronically ill children, 48% had exclusively ill children, while 52% cared for both chronically ill and healthy children. Chronic diseases included gastroenterological and hepatological diseases in 29%, respiratory illnesses in 26%, metabolic conditions in 9%, hemato-oncological diseases in 8%, and renal and rheumatological diseases in 6% of all cases each.

**Table 1:** Caregivers’ characteristics

**Caregivers’ characteristics**	**Caregivers with chronically ill children (*n* = 128)**		**Caregivers with healthy children (*n* = 77)**	
	***n***	**%**	***n***	**%**
**Gender**				
Female/male	96/32	75/25	60/17	78/22
**Age [years]**				
16–20	0	0	1	1
21–30	6	5	9	12
31–40	56	44	32	42
41–50	50	39	29	38
51–60	14	11	6	8
61–70	2	2	0	0
**Caregivers with an occupation in the healthcare system**				
Yes/no/unknown	30/97/1	23/76/1	12/65/0	16/84/0
**Children age [years]**				
<2	25	9	22	15
2–5	58	21	39	26
6–12	103	37	47	31
13–18	65	24	30	20
>18	25	9	12	8
**COVID-19 cases in children**				
Yes/no/unknown	3/49/76	2/38/59	4/17/56	5/22/73
**COVID-19 cases within the family**				
Yes/no/unknown	6/119/3	5/93/2	5/71/1	6/92/1
**COVID-19 cases within the peer group**				
Yes/no/unknown	75/52/1	59/41/1	42/32/3	55/42/4

Since, as expected, more mothers visit the outpatient department with their children, a significantly higher proportion of female caregivers (76%) than male caregivers (24%) participated in the questionnaire (Table 1). The majority of participants (81%) were between 31 and 50 years old, while the most commonly reported age range for their children was 6 to 12 years of age, with 37% of chronically ill children and 31% of healthy children falling within this range (Table 1). The percentage of children who reported previous COVID-19 infection was low, with 2% of chronically ill and 5% of healthy children having contracted the virus (Table 1).

### Concerns of contracting COVID-19

**Fig. 1:**
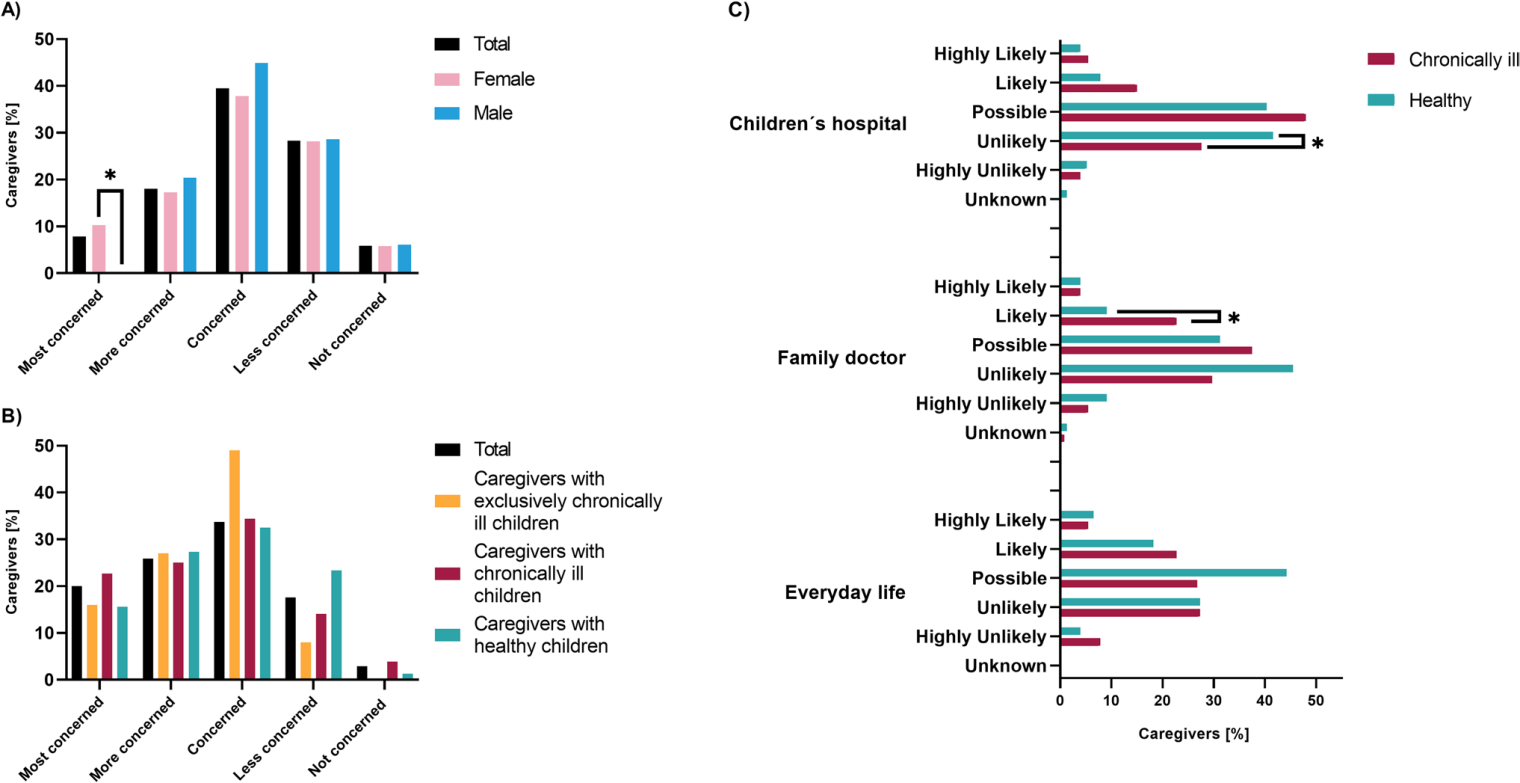
Concerns of contracting COVID-19 in caregivers of chronically ill and healthy children. **A** Concerns of caregivers regarding their own risk of contracting COVID-19. **B** Concerns of caregivers regarding COVID-19 of their children, distinguished by caregivers with and without chronically ill children. **C** Caregivers evaluation of SARS-CoV-2 transmission risk in different settings, distinguishing between caregivers with and without chronically ill children. Group differences were analyzed by chi-square test (*p < 0.05)

We first asked caregivers about their own concerns of contracting COVID-19 themselves. Using a 5-level Likert scale describing a range from “not concerned” to “most concerned,” 26% of all caregivers stated they were “most or more concerned” of contracting SARS-CoV-2 themselves with female caregivers being significantly overrepresented in the group “most concerned” (*n* = 205; *w* = 0.16; *p* = 0.02; Fig. 1A). When caregivers had experienced COVID-19 within their peer group, they were significantly more likely to be concerned (*n* = 201; *p* = 0.02).

Asked for concerns about their children contracting SARS-CoV-2, 45.9% of all participants stated they were “most or more concerned” (Fig. 1B). In caregivers with chronically ill children, 22.8% were “most concerned” of a SARS-CoV-2 infection of their children compared to 15.6% in caregivers with healthy children (*n* = 205; not significant, Fig. 1B). Participants with exclusively chronically ill children were not more concerned than participants with healthy children (*n* = 114; not significant, Fig. 1B). Of note, caregivers of children with hemato-oncological diseases (*n* = 10) were more concerned about their child becoming infected with COVID-19 than were caregivers of other (*n* = 194) patient groups (*p* = 0.028).

We further analyzed caregivers’ perceived risk for their children in which setting to contract SARS-CoV-2: Caregivers considered a likely or highly likely risk of SARS-CoV-2 transmission at a children’s hospital, at their family doctor, and in everyday life situations in 62.3%, 56.6%, and 66.4%, respectively (Fig. 1C). Caregivers of chronically ill children considered the risk of transmission both at the family doctor and at a children’s hospital to be significantly more “likely” than caregivers of healthy children (*n* = 203; *p* = 0.04 and *n* = 205; *p* = 0.04, respectively; Fig. 1C). In sum, caregivers of chronically ill children considered a higher risk to contract SARS-CoV-2 at healthcare settings.

### Caregivers-reported HRQoL and overall QoL

**Fig. 2:**
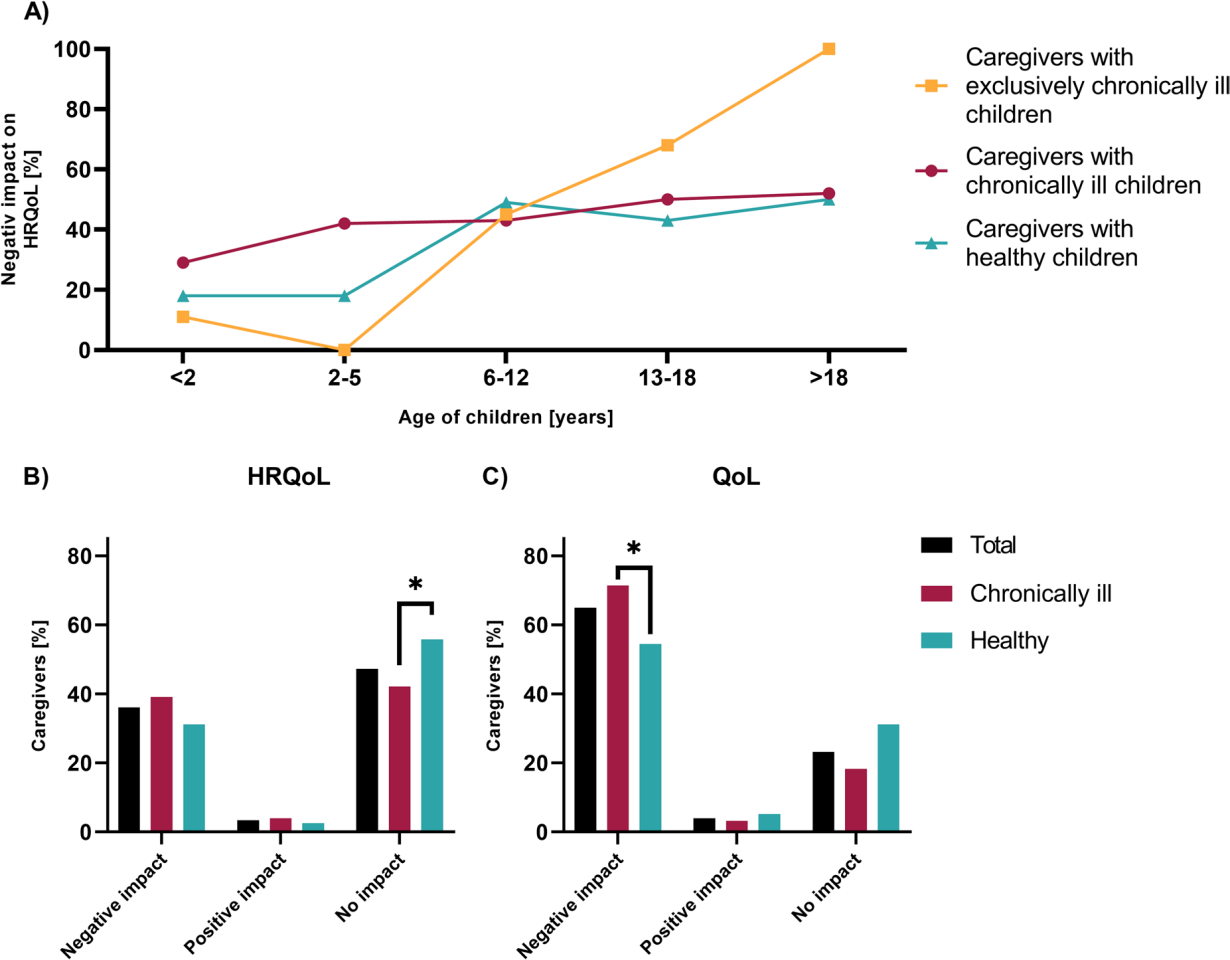
Caregivers-reported HRQoL and QoL of chronically ill compared to healthy children during the COVID-19 pandemic. **A** Percentage of caregivers reporting a negative impact of the COVID-19 pandemic on the HRQoL of their children, differentiated by children’s age and comparing caregivers with exclusively chronically ill, chronically ill, and without chronically ill children. **B** Percentage of caregivers reporting a negative or positive impact of the COVID-19 pandemic on the HRQoL of their children, comparing participants with and without chronically ill children. **C** Percentage of caregivers reporting a negative or positive impact of the COVID-19 pandemic on the QoL of their children, comparing caregivers with and without chronically ill children. Group differences were analyzed by chi-square test (*p < 0.05)

While some studies assessed HRQoL or QoL of caregivers from either healthy or chronically ill children and adolescents [14–16, 22], we aimed to analyze and compare differences of both groups. 35.4% of all participants reported of a negative impact of the COVID-19 pandemic on HRQoL of their children, while 3.6% indicated a positive impact (Fig. 2B). Forty percent of caregivers of chronically ill children and 31.2% of caregivers of healthy children declared a negative impact of COVID-19 on the HRQoL of their children (Fig. 2A and B). Caregivers with exclusively chronically ill adolescents over the age of 13 declared a significantly stronger negative impact on their children’s HRQoL compared to caregivers of healthy adolescents in this age group (*n* = 79; *w* = 0.19; *p* = 0.016; Fig. 2A). Of note, all caregivers reported an increasing negative impact on the HRQoL with rising age of their children, with 100% of caregivers of exclusively chronically ill adolescents over the age of 18 years (Fig. 2A). Caregivers of healthy children stated significantly more often that the COVID-19 pandemic had no impact on the HRQoL of their children than caregivers of chronically ill children (*n* = 203; *w* = 0.18; *p* = 0.043; Fig. 2B).

Assessing the overall QoL, 65.0% of all caregivers reported a negative impact of the COVID-19 pandemic on their children, while only 3.9% indicated a positive impact (Fig. 2C). Caregivers of chronically ill children significantly more often declared a negative impact of the COVID-19 pandemic on their fosterlings overall QoL compared to caregivers of healthy children (*n* = 203; *w* = 0.17; *p* = 0.014; Fig. 2C).

### Healthcare utilization of chronically ill compared to healthy children during the COVID-19 pandemic

**Fig. 3:**
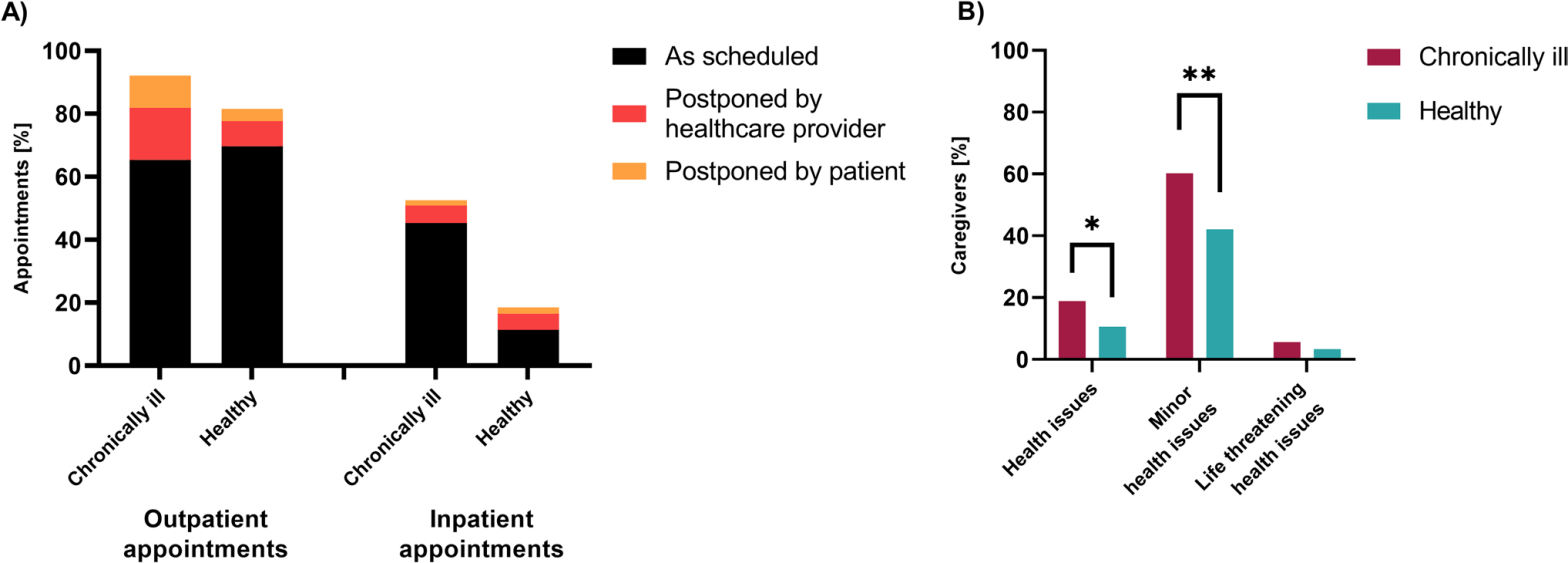
Healthcare utilization of caregivers from chronically ill compared to healthy children during the COVID-19 pandemic. **A** Percentage of medical appointments (if scheduled) taken place as scheduled or postponed by the healthcare provider or the patient, differentiating outpatient and inpatient appointments as well as children with and without chronic disease. Patients who did not have an appointment are not indicated and represent the missing percentages. **B** Percentage of caregivers reporting they would delay medical visits for health issues in general, for minor health issues, and for life-threatening health issues due to the COVID-19 pandemic, comparing caregivers with and without chronically ill children. Group differences were analyzed by chi-square test (*p < 0.05; **p < 0.01)

Although first studies are available analyzing the access to and utilization of healthcare for adult patients with and without chronic diseases [23–25], data on children and adolescents is limited [16, 26, 27] while no study compared between healthy and chronically ill children, so far. Of all caregivers assessed in our study, 91.3% reported their children having regular medical appointments (Fig. 3A). Postponing or cancelation—either by the family or the hospital—of appointments for children with a chronical illness including diagnostic and therapeutic services and surgery due to the pandemic was stated in 29.7% for outpatient and in 13.4% for inpatient visits (Fig. 3A). This was reported significantly more often in chronically ill children than from caregivers of healthy children (*n* = 180; *w* = 0.17; *p* = 0.025). For these otherwise healthy children, preventive check-ups or elective surgery was canceled or postponed in 14.5% of outpatient and 7.1% of inpatient appointments (Fig. 3A).

8.9% of 108 caregivers with a chronical ill child on long-term medication reported difficulties in obtaining this medication due to the COVID-19 pandemic. 30.7% of the families with chronically ill children stated their wish for telehealth-based methods in the healthcare system (e.g., video or phone calls). 41.1% of the families with a chronical ill child declared that there was no information provided to them about the COVID-19 pandemic and the specific risks for their children. Moreover, 22.6% of the families would have preferred availability of more information regarding their specific COVID-19 risks and the necessary safety precautions.

Caregivers stated significantly more often that they would delay medical consultation for a new-onset health issue for their chronically ill child than for healthy children due to fear of contracting COVID-19 (*n* = 278; *w* = 0.12; *p* = 0.049; Fig. 3B). Especially for minor health issues (described as for example a cough or diarrhea), caregivers would delay medical visits for their chronically ill children significantly more often than they would do with their healthy children (*n* = 278; *w* = 0.18; *p* = 0.003; Fig. 3B).

Thus, the perceived access (measured by postponements or cancelation of appointments) and the utilization of healthcare services were more limited for children with chronic disease compared to healthy children during the COVID-19 pandemic.

## Discussion

The COVID-19 pandemic and the associated containment measures had a severe impact on everyday life of families and children. As many hospitals limited their services, disruption of healthcare is especially challenging for children with a chronic underlying disease and substantial medical demands. Furthermore, chronical ill patients could be at risk for a severe disease course when contracting COVID-19. These circumstances put children with a chronic disease at risk for psychosocial and health impairments. Recent publications have evaluated the impact of the COVID-19 pandemic on chronically ill compared to healthy adults [28–30] and analyzed the differences in HRQoL before and during the pandemic in children with specific chronic diseases [31, 32]. However, this is the first study evaluating and comparing the caregivers’ concerns of COVID-19 for their children, the perceived impact on quality of life of their fosterlings, and the utilization of healthcare services in both, families caring for chronically ill children and families with healthy children.

Almost 50% of our questionnaire participants expressed major concerns about their children contracting SARS-CoV-2. This is in line with data presented by Nicholson et al., reporting 41% of parents of healthy children being majorly concerned about their children contracting COVID-19 [33]. Since our study was conducted spanning the second SARS-CoV-2 infection wave in Germany when the COVID-19-related death rate was drastically rising while a vaccination was not yet available for the broad population, at this time period the general level of uncertainty and fear from infection was extraordinarily high [18, 20]. Notably, we found no significant difference between the level of concern in families with chronically ill versus healthy children. Compared to our more heterogenous group of different chronic diseases in this study, Darlington et al. reported 85.4% of parents being concerned about their children contracting COVID-19 in a specific cohort of children suffering from cancer [34]. This is in line with our finding that guardians of children with a hemato-oncological condition were more concerned about their child contracting COVID-19 than were caregivers of other patient groups. Such differences may be explained by disease group specificities, current disease status such as higher perceived infection risks in cancer patients under chemotherapy as well as differences in public discussion and media coverage. To capture this aspect systematically, a larger cohort group with a detailed questionnaire would be required. We show that caregivers of chronically ill children evaluated the risk of a SARS-CoV-2 infection at the children’s hospital significantly less as “unlikely.” Darlington et al. support these findings by reporting a high-risk evaluation of COVID-19 transmission in the hospital by parents with children suffering from cancer [34]. This knowledge should be addressed in patient guidance through the hospital and outpatient clinic as well as in isolation considerations.

While there are multiple studies assessing the HRQoL of children and adolescents during the COVID-19 pandemic [22, 35], only a few of those are focusing on HRQoL of chronically ill children [31, 32]. In our study, 37% of all participants reported that the COVID-19 pandemic and the accompanying restrictive measures had a negative impact on their children’s health-related quality of life. Rihm et al. demonstrated a reduced caregivers-reported HRQoL in children with rare diseases during the COVID-19 pandemic [31]. In our study, for adolescents over 13 years of age but not for younger children, caregivers of chronically ill adolescents reported negative effects on HRQoL significantly more often than caregivers of healthy adolescents. This age dependency may be explained by more comprehensive impairment of social needs in an older child due to social distancing and school closure which could further be multiplied by the presence and respective burden of a chronic disease. This may be supported by the findings of Aman et al. who showed in 2022 that higher age of children with nephrotic syndrome was related with greater sleep disruption and therefore a decline in HRQoL during the COVID-19 pandemic [32]. When assessing caregivers-reported non-health-related QoL, over 50% of healthy children were reported that the COVID-19 pandemic and its associated restrictions had a negative impact. Several other studies assessing healthy children indicated similar results [35, 36]. Chronically ill children in our study cohort were reported to be significantly more often negatively impacted in their QoL due to the COVID-19 pandemic. This is in line with Onal et al., who reported an increase of treatment anxiety, lower satisfaction scores, and decrease of QoL in pediatric cancer patients during the COVID-19 pandemic [37].

Chronically ill children are dependent on continuous medical care and have regular follow-up and multidisciplinary visits. Healthy children usually only present for screening check-ups or with acute health issues. Alarmingly, our data indicated that medical appointments of chronically ill children were significantly more often postponed or canceled, although Remppis et al. showed that cancelation of routine appointments was perceived as a particular burden by healthcare providers while not being a suitable measure to prevent SARS-CoV-2 transmission [38]. Considering only the inpatient appointments, for which patients had a planned admission to a hospital ward for a diagnostic or interventional procedure, we show that almost as many appointment delays or cancelations were initiated by the patients’ families as were by the healthcare providers. Therefore, the combination of hospitals reducing healthcare services and caregivers avoiding healthcare utilization due to the fear of contracting COVID-19 points to a multi-faceted problem in healthcare for chronically ill children during a pandemic. Of note, caregivers of chronically ill children reported significantly more often to be willing to delay medical appointments in case of health issues. At the time of our study in the pandemic, the severity of COVID-19 for children and adolescents was still unclear but first reports assigned both adults and children with certain pre-existing conditions as higher risk groups for severe COVID-19 [39, 40]. Further, the German population’s fear from infection was presumable very high during that time which we could confirm for the participants we analyzed (Fig. 1). These facts could have provoked an increased avoidance behavior of caregivers of chronically ill children. Avoidance of medical facilities due to concerns of SARS-CoV-2 transmission has been shown in multiple other studies, including parents with healthy children [33] as well as parents with chronically ill children [34]. Remppis et al. demonstrated a considerable drop of emergency department visits, as well as hospital admissions at a tertiary care children’s hospital during the first year of the COVID-19 pandemic compared to previous years [38]. It is noteworthy that a few of the families with chronically ill and with healthy children stated that they would even delay seeking medical help in case of a life-threatening emergency. This is supported by the findings of Neill et al. in 2021, who illustrated in a retrospective survey that parents of healthy children did not always seek medical help even when their children were showing severe symptoms [41]. We therefore highlight the need to strongly instruct caregivers to utilize the healthcare system irrespective of pandemics, such as COVID-19, especially considering that children and adolescents experience mostly mild symptoms and are not in the risk group for severe COVID-19 infections [3]. Contactless digital healthcare options could counteract avoidance behavior of caregivers and therefore support good patient care, with 30.7% of our participants with chronically ill children stating to prefer contactless healthcare options during the COVID-19 pandemic. Mercuri et al. described a personalized telehealth model specifically designed for the treatment and follow-up examinations of chronic pediatric diseases [42]. The implementation of telehealth care services during the COVID-19 era is anticipated to become a permanent part of the healthcare system, providing long-term benefits, particularly for patients with chronic conditions who require frequent medical appointments and are at a higher risk of germ transmission [43] and chronically ill patients living in remote areas. 22.6% of the families with chronically ill children would have appreciated more specific information about the risks and precautions necessary for their children. The high emotional burden of families with chronically ill children as well as the caregivers’ avoidance behavior towards healthcare during the pandemic could be minimized through appropriate delivery of disease-specific information by healthcare providers.

There were limitations to this study. It was conducted at a large academic tertiary care children’s hospital and may therefore not be representative for other hospitals. The study could only be conducted in participants that were actually presenting in our hospital. Families who avoided hospital visits due to fear of infection could not be included in the study. Further, families that were not able to speak and read German meeting at least reference level B2 defined by the CEFR were excluded as they were not able to fully understand the questionnaire. The measured results could therefore even underestimate the multi-faceted problem of healthcare utilization and quality of life. There could be some additional unmeasured confounding factors (e.g., specific chronic disease, treatment intensity, disease severity) that may affect the results of the survey. The study measured the implications of COVID-19 only between October 2020 and March 2021, missing potential developments over the long-lasting course of the pandemic and the different restrictions at various time points. The study does not use validated questionnaires to evaluate the HRQoL, like the SF-36 [44], but rather single questions, not accounting for the different aspects of HRQoL which is proxy-reported in our study. Furthermore, the study does not explore HRQoL, other quality of life or healthcare utilization before the COVID-19 pandemic, denying the possibility of pre- and post-pandemic comparisons. Nevertheless, the consistency of findings of this study with the results from multiple other studies suggests that the results can be applied to a larger cohort.

## Conclusions

According to their caregivers, chronically ill children suffered significantly more often from negative impacts of the COVID-19 pandemic on their quality of life than healthy children. Their medical appointments were more frequently postponed or canceled and parents were significantly more willing to delay medical consultations than parents of healthy children. In exceptional situations like the COVID-19 pandemic and its associated restrictions, it is therefore indispensable to particularly support families with chronically ill children in their specific concerns and needs. This could be achieved by reliable appointment allocation by the healthcare provider, offering contactless healthcare options, as well as frequently delivering information tailored to susceptibilities and precautions specifically necessary for their children.

### Supplementary Information

Below is the link to the electronic supplementary material.

Supplementary file1 (PDF 224 KB)

Supplementary file2 (PDF 222 KB)

## Abbreviations

COVID-19: Corona virus infectious disease 2019
HRQoL: Health-related quality of life
SARS-CoV-2: Severe acute respiratory syndrome coronavirus 2
QoL: Quality of Life
