# Supplementary material for: Quality of life and healthcare utilization during the COVID-19 pandemic are more restricted in chronically ill than in healthy children: a tertiary care children’s hospital experience
Source: Eur J Pediatr. 2024 Jan 23;183(4):1801–10. doi: 10.1007/s00431-023-05382-6 (PMC11001739; doi:10.1007/s00431-023-05382-6)
Supplement: Supplementary file 1 — Supplementary file1 (PDF 224 KB) [file 431_2023_5382_MOESM1_ESM.pdf]

Fragebogen

Datum:
No.:

Bitte vollständig ausfüllen und nur jeweils ein Antwortkreuz setzen. Bei Rückfragen wenden Sie sich gerne an einen der Studienärzte.

Dauer: ~ 10 Minuten

Einverständnis zur Teilnahme:

Vielen Dank, dass Sie sich bereiterklären an oben genannter Umfragestudie teilzunehmen. Fand eine ausreichende Aufklärung statt, wurden Ihre Fragen beantwortet und wollen Sie an oben genannter Umfragestudie teilnehmen?

Ja ☐	Nein ☐

Abschnitt „1“ – Teilnehmer

1. Sind Sie Elternteil oder Sorgeberechtigte/r, der das Kind/die Kinder heute begleitet?

Elternteil ☐	Sorgeberechtigte/r ☐	Nicht zutreffend bzw. Keine Angabe ☐

1.1 Welcher Altersgruppe gehören Sie an?

16-20 Jahre ☐	21-30 Jahre ☐	31-40 Jahre ☐	41-50 Jahre ☐	51-60 Jahre ☐	61-70 Jahre ☐	> 70 Jahre ☐

1.2 Welches Geschlecht haben Sie?

Männlich ☐	Weiblich ☐	Divers ☐

1.3 Was ist ihr Beruf?

Student/in ☐	Wissenschaftlicher Mitarbeiter/in ☐	Angestellte/r ☐	Selbstständige Arbeit ☐	Arbeitssuchend ☐	Rentner/in ☐	Nicht zutreffend bzw. Keine Angabe ☐

1.4 Sind Sie im Gesundheitswesen tätig? (z.B. Pflege, Ärztin/Arzt, Med. Fachangestellte/r)

Ja ☐	Nein ☐	Keine Angabe ☐

Abschnitt „2“ – Neuartiges Corona-Virus / Corona-Pandemie

2.1 Waren Sie oder andere Personen, die mit Ihrem Kind / Ihren Kindern in einem Haushalt leben an COVID-19 erkrankt? (Achtung: Die Kinder selbst sind nicht gemeint!)

Ja, mit positivem Test ☐	Ja, ohne Test ☐	Nein, mit negativem Test ☐	Nein, ohne Test ☐	Weiß nicht / Keine Angabe ☐

2.2 Kennen Sie jemanden der an COVID-19 erkrankt ist / war oder arbeiten Sie mit Menschen, die an COVID-19 erkrankt sind / waren?

Ja ☐	Nein ☐	Weiß nicht / Keine Angabe ☐

2.3 Wie groß ist Ihre Sorge selbst an COVID-19 zu erkranken?

Sehr groß ☐	Groß ☐	Moderat ☐	Klein ☐	Gar nicht ☐	Weiß nicht / Keine Angabe ☐

2.4 Wie groß ist Ihre Sorge, dass Ihr(e) Kind(er) an COVID-19 erkrankt / erkranken?

Sehr groß ☐	Groß ☐	Moderat ☐	Klein ☐	Gar nicht ☐	Weiß nicht / Keine Angabe ☐

Die nun folgenden Fragen 2.5 – 2.9 beziehen sich auf den Zeitraum der gesamten Corona-Pandemie!

2.5 Wie groß schätzen Sie das Risiko ein, dass Ihr(e) Kind(er) sich bei Besorgungen des Alltags (z.B. Einkaufen im Supermarkt, Besuch eines Spielplatzes) ansteckt / anstecken?

Sehr groß ☐	Groß ☐	Moderat ☐	Klein ☐	Gar nicht ☐	Weiß nicht / Keine Angabe ☐

2.6 Wie groß schätzen Sie das Risiko ein, dass Ihr(e) Kind(er) sich bei einem Besuch beim <u>niedergelassenen Kinderarzt / Hausarzt</u> mit dem Corona-Virus ansteckt / anstecken?

Sehr groß ☐	Groß ☐	Moderat ☐	Klein ☐	Gar nicht ☐	Weiß nicht / Keine Angabe ☐

2.7 Wie groß schätzen Sie das Risiko ein, dass Ihr(e) Kind(er) sich bei einem Besuch <u>in einer Kinderklinik</u> mit dem Corona-Virus ansteckt / anstecken?

Sehr groß ☐	Groß ☐	Moderat ☐	Klein ☐	Gar nicht ☐	Weiß nicht / Keine Angabe ☐

2.8 Hat die Corona-Pandemie die *<u>psychische und / oder körperliche</u>* Gesundheit Ihres/r Kind(er) beeinflusst?

Ja, negativ beeinflusst ☐	Ja, positiv beeinflusst ☐	Nein, nicht beeinflusst ☐	Weiß nicht / Keine Angabe ☐

2.9 Hat die Corona-Pandemie die *<u>sonstige</u>* Lebensqualität Ihres/r Kind(er) beeinflusst?

Ja, negativ beeinflusst ☐	Ja, positiv beeinflusst ☐	Nein, nicht beeinflusst ☐	Weiß nicht / Keine Angabe ☐

Abschnitt „3" – Kind(er)

3.1 Wie viele Kinder haben Sie?

1 Kind ☐	2 Kinder ☐	3 Kinder ☐	4 Kinder ☐	> 4 Kinder ☐	Keine Angabe ☐

3.2 Wie alt sind die Kinder? (Hier sind Mehrfachantworten möglich!)

Kind	0-1 Jahr (Bedeutung: innerhalb der ersten 24 Lebensmonate)	2-5 Jahre	6-12 Jahre	13-18 Jahre	>18	Keine Angabe
#1	☐	☐	☐	☐	☐	☐
#2	☐	☐	☐	☐	☐	☐
#3	☐	☐	☐	☐	☐	☐
#4	☐	☐	☐	☐	☐	☐
#5	☐	☐	☐	☐	☐	☐
#6	☐	☐	☐	☐		☐
#7	☐	☐	☐	☐		☐

3.3 Hat eines Ihrer Kinder eine chronische Erkrankung (z.B. Asthma, Neurodermitis, chronisch entzündliche Darmerkrankung, Rheuma, etc., siehe Definition)?

Ja ☐<br>➔ **Ab Abschnitt 4 weiter ausfüllen (weiter ab Seite 5 bis Ende)**	Nein ☐<br>➔ ***Nur* ab Abschnitt 5 weiter ausfüllen (ab Seite 9 bis Ende)**

Definition chronischer Erkrankung (im Kindesalter):

- Erkrankung / Zustand besteht seit min. 3 Monaten (Achtung: Kinder unter 1 Jahr hier nicht zu bewerten)[1]
- Erkrankung / Zustand schränkt altersangemessene Aktivitäten ein und / oder hat wiederkehrenden Bedarf einer medizinischen Versorgung / Kontrolluntersuchung[1]
- *Generell gilt: Eine Diagnose durch den Kinderarzt die über einen längeren Zeitraum als 3 Monate besteht gilt als chronische Erkrankung*

[1]Schmidt, Silke & Thyen, Ute. (2008). Was sind chronisch kranke Kinder?. Bundesgesundheitsblatt - Gesundheitsforschung - Gesundheitsschutz. 51. 585-591. 10.1007/s00103-008-0534-5.

3.3.1 Falls „JA", mit welchem Ihrer Kinder stellen Sie sich heute mit Termin oder einer akuten Erkrankung in der Kinderklinik vor?

Mein(e) chronisch krankes/en Kind(er) ☐	Mein(e) sonst gesunden Kind(er) ☐

Abschnitt „4“ – Familien mit chronisch kranken Kindern / Vorgehen mit chronisch kranken Kindern

4.1 Wie viele Ihrer Kinder haben eine chronische Erkrankung?

**1 Kind ☐**	**2 Kinder ☐**	**3 Kinder ☐**	**4 Kinder ☐**	**> 4 Kinder ☐**	**Keine Angabe ☐**

4.2 Um was für eine Art der chronischen Erkrankung handelt es sich? (Hier sind Mehrfachantworten möglich!)

**Kind**	**Atemwege** (z.B. Asthma)	**Leber und/oder Darm**	**Herz**	**Niere**	**Rheuma /Gelenke**	**Hämatologie /Onkologie** (z.B. Krebserkrankung)	**Stoffwechsel** (z.B. Diabetes mellitus)	**anderes**	**Keine Angabe / weiß nicht**
**#1**	☐	☐	☐	☐	☐	☐	☐	☐	☐
**#2**	☐	☐	☐	☐	☐	☐	☐	☐	☐
**#3**	☐	☐	☐	☐	☐	☐	☐	☐	☐
**#4**	☐	☐	☐	☐	☐	☐	☐	☐	☐
**#5**	☐	☐	☐	☐	☐	☐	☐	☐	☐
**#6**	☐	☐	☐	☐	☐	☐	☐	☐	☐
**#7**	☐	☐	☐	☐	☐	☐	☐	☐	☐

4.3 War (oder ist) ihr chronisch krankes Kind / Kinder an COVID-19 erkrankt?

Ja, mit positivem Test ☐	Ja, ohne Test ☐	Nein, mit negativem Test ☐	Nein, ohne Test ☐	Weiß nicht / Keine Angabe ☐

4.4 Nimmt eines Ihrer chronisch kranken Kinder aufgrund seiner Erkrankung regelmäßig/dauerhaft Medikamente ein?

Ja ☐	Nein ☐	Weiß nicht / Keine Angabe ☐

4.4.1 Falls „JA“, kam es aufgrund der Corona-Pandemie zu Versorgungsproblemen mit den benötigten Medikamenten?

Ja ☐	Nein ☐	Weiß nicht / Keine Angabe ☐

4.5 Macht die chronische Erkrankung eines / mehrerer Ihrer Kinder regelmäßige ambulante Arztbesuche und / oder stationäre Aufnahmen in einer Klinik notwendig?

Ja ☐	Nein ☐	Weiß nicht / Keine Angabe ☐

4.6 Kam es aufgrund der Corona-Pandemie zu Terminverschiebungen oder -ausfällen von ambulanten Vorstellungen bei Ihrer Kinderarzt/ Hausarzt oder in einer Spezialsprechstunde (z.B. in einer Kinderklinik) für eines Ihrer chronisch kranken Kinder?

Ja, die Klinik/Praxis hat den Termin abgesagt ☐	Ja, ich/wir haben den Termin abgesagt ☐	Nein, keine Terminverschiebung ☐	Nein, kein Arztbesuch war geplant / erforderlich ☐	Weiß nicht / Keine Angabe ☐

4.6.1 Falls „JA“: Wo war dieser Termin geplant?

Niedergelassener Kinderarzt / Hausarzt ☐	Spezialsprechstunde (z.B. Asthmasprechstunde in einer Kinderklinik) ☐	Ambulante Intervention / Diagnostik (z.B. ambulante OP, Bildgebung wie Röntgen/CT/MRT, etc.) ☐	Sonstiges (z.B. Physiotherapie, Reha, etc.) ☐	Weiß nicht / Keine Angabe ☐

4.6.2 Falls „JA“, waren davon notwendige Termine für reguläre Vorsorgeuntersuchungen (U-Untersuchungen) oder Impfungen betroffen?

Ja ☐	Nein ☐	Weiß nicht / Keine Angabe ☐

4.6.3 Falls „JA, ich/wir haben den Termin abgesagt“: Haben Sie aus Sorge vor einer Ansteckung mit dem Corona-Virus den ambulanten Termin abgesagt oder verschoben?

Ja ☐	Nein ☐	Weiß nicht / Keine Angabe ☐

4.6.4 Falls „JA, die Klinik/Praxis hat den Termin abgesagt“, wurden als Ersatz für die Vorstellung in der Spezialsprechstunde alternative Möglichkeiten angeboten, wie z.B. Videosprechstunde, Telefonsprechstunde, ausführliche Telefonate o.ä.?

Ja ☐	Nein ☐	Weiß nicht / Keine Angabe ☐

4.7 Haben Sie das Angebot einer kontaktlosen medizinischen Versorgung wie Videosprechstunde, Telefonsprechstunde, ausführliche Telefonate oder Ähnliches. genutzt?

Ja ☐	Nein ☐	Weiß nicht / Keine Angabe ☐

4.7.1 Falls „JA“, wie gut konnte dies die persönliche Vorstellung in der Spezialsprechstunde ersetzen?

Sehr gut ☐	Gut ☐	Moderat ☐	Wenig ☐	Gar nicht ☐	Weiß nicht / Keine Angabe ☐

4.8 Wünschen Sie sich im Rahmen der Corona-Pandemie mehr Angebote einer kontaktlosen medizinischen Versorgung, wie z.B. Videosprechstunde, Telefonsprechstunde, ausführliche Telefonate oder Ähnliches?

Ja ☐	Nein ☐	Weiß nicht / Keine Angabe ☐

4.9 Kam es aufgrund der Corona-Pandemie zu Terminverschiebungen oder -ausfällen von <u>stationären Terminen</u> für eines Ihrer chronisch kranken Kinder?

Ja, die Klinik/Praxis hat den Termin abgesagt ☐	Ja, ich/wir haben den Termin abgesagt ☐	Nein, nicht verschoben ☐	Nein, es waren <u>keine</u> stationären Termine geplant ☐	Weiß nicht / Keine Angabe ☐

4.9.1 Falls „JA, ich/wir haben den Termin abgesagt“, haben Sie aus Sorge vor einer Ansteckung mit dem Corona-Virus stationäre Termine abgesagt oder verschoben?

Ja ☐	Nein ☐	Weiß nicht / Keine Angabe ☐

4.10 Falls Terminabsagen oder -verschiebungen von ambulanten <u>und/oder</u> stationären Terminen stattfanden:
Wie sehr hat dies *aus Ihrer Sicht* die Gesundheit Ihres Kindes <u>negativ</u> beeinträchtigt?

Sehr stark ☐	Stark ☐	Moderat ☐	Wenig ☐	Gar nicht ☐	Weiß nicht / Keine Angabe ☐	Nicht zutreffend / Keine Terminabsagen ☐

4.11 Haben die behandelnden Ärzte / Therapeuten Ihnen Informationen zum Umgang mit dem Corona-Virus bei ihrem chronischen kranken Kind / Kindern zur Verfügung gestellt?

Ja, ausreichend Informationen ☐	Ja, aber zu wenig Informationen ☐	Nein, keine Informationen ☐	Weiß nicht / Keine Angabe ☐

4.11.1 Haben / Hätten sie <u>mehr</u> Bedarf an (z.B. online zugänglichen) Informationen zum Umgang mit dem Corona-Virus bei Ihrem chronisch kranken Kind / Kindern gehabt?

Ja ☐	Nein ☐	Weiß nicht / Keine Angabe ☐

4.12 Hat die Corona-Pandemie insgesamt aus Ihrer Sicht Auswirkungen auf die <u>Gesundheit</u> Ihres chronisch kranken Kindes / meiner chronisch kranken Kinder?

Ja, negative Auswirkungen ☐	Ja, positive Auswirkungen ☐	Nein, keine Auswirkungen ☐	Weiß nicht / Keine Angabe ☐

4.13 Würden Sie im Falle einer akuten Erkrankung *generell* bei einem Ihrer Kinder einen Arztbesuch während der Corona-Pandemie, vermeiden oder hinauszögern?

Ja ☐	Nein ☐	Weiß nicht / Keine Angabe ☐

4.14 Würden Sie im Falle einer akuten Erkrankung *aber für Sie leichten Problematik* *(z.B. Husten, Durchfall)* bei einem Ihrer Kinder einen Arztbesuch während der Corona-Pandemie, vermeiden oder hinauszögern?

Ja ☐	Nein ☐	Weiß nicht / Keine Angabe ☐

4.15 Würden Sie im Falle einer akuten, *für Sie möglicherweise lebensbedrohlichen* Erkrankung bei einem Ihrer Kinder einen Arztbesuch während der Corona-Pandemie, vermeiden oder hinauszögern?

Ja ☐	Nein ☐	Weiß nicht / Keine Angabe ☐

Abschnitt „5“ – Familien mit sonst gesunden Kinder / Vorgehen bei gesunden Kindern

BITTE BEACHTEN:

Falls Sie nur chronisch kranke Kinder / ein chronisch krankes Kind haben bitte NICHT ausfüllen!

5.1 War (oder ist) ihr sonst gesundes Kind / Kinder an COVID-19 erkrankt?

Ja, mit positivem Test ☐	Ja, ohne Test ☐	Nein, mit negativem Test ☐	Nein, ohne Test ☐	Weiß nicht / Keine Angabe ☐

5.2 Kam es aufgrund der Corona-Pandemie zu Terminverschiebungen oder -ausfällen beim Kinderarzt / Hausarzt für eines Ihrer Kinder?

Ja, die Klinik/Praxis hat den Termin abgesagt ☐	Ja, ich/wir haben den Termin abgesagt ☐	Nein, keine Terminverschiebung ☐	Nein, kein Arztbesuch war geplant / erforderlich ☐	Weiß nicht / Keine Angabe ☐

5.2.1 Falls „JA“, welche Art von Vorstellung war geplant?

Reguläre Vorsorgeuntersuchungen (z.B. U-Untersuchungen, Impfungen, etc.) ☐	Akute Vorstellung (z.B. bei Atemwegsinfektion, Bauchschmerzen, etc.) ☐	Sonstiges (z.B. Abholung eines Rezepts, Entschuldigung für den Schulunterricht ☐	Keine Angabe ☐

5.2.2 Falls „JA, ich/wir haben den Termin abgesagt“, haben Sie einen oder mehrere Termine *aus Sorge vor Ansteckung mit dem Corona-Virus* verschoben oder abgesagt?

Ja ☐	Nein ☐	Weiß nicht / Keine Angabe ☐

5.2.3 Falls „JA“, wie sehr hat die Terminverschiebung oder -ausfall die Gesundheit Ihres Kindes aus Ihrer Sicht negativ beeinträchtigt?

Sehr stark ☐	Stark ☐	Moderat ☐	Wenig ☐	Gar nicht ☐	Weiß nicht / Keine Angabe ☐

5.3 Sollte eines Ihrer Kinder während der Corona-Pandemie stationär in einem Krankenhaus aufgenommen werden?

Ja ☐	Nein ☐	Weiß nicht / Keine Angabe ☐

5.3.1 Falls „JA“, haben Sie die stationäre Aufnahme Ihres Kindes aus Sorge vor COVID-19 auf eigenen Wunsch abgelehnt oder haben auf eigenen Wunsch das Krankenhaus früher als geplant verlassen?

Ja ☐	Nein ☐	Weiß nicht / Keine Angabe ☐

5.3.1.1 Falls „JA“, wie sehr hat dies aus Ihrer Sicht die Gesundheit Ihres Kindes negativ beeinträchtigt?

Sehr stark ☐	Stark ☐	Moderat ☐	Wenig ☐	Gar nicht ☐	Weiß nicht / Keine Angabe ☐

5.4 Würden Sie im Falle einer akuten Erkrankung *<u>generell</u>* bei einem Ihrer Kinder einen Arztbesuch während der Corona-Pandemie, vermeiden oder hinauszögern?

Ja ☐	Nein ☐	Weiß nicht / Keine Angabe ☐

5.5 Würden Sie im Falle einer akuten Erkrankung *<u>aber für Sie leichten Problematik</u> (z.B. Husten, Durchfall)* bei einem Ihrer Kinder einen Arztbesuch während der Corona-Pandemie, vermeiden oder hinauszögern?

Ja ☐	Nein ☐	Weiß nicht / Keine Angabe ☐

5.6 Würden Sie im Falle einer akuten, *<u>für Sie möglicherweise lebensbedrohlichen</u>* Erkrankung bei einem Ihrer Kinder einen Arztbesuch während der Corona-Pandemie, vermeiden oder hinauszögern?

Ja ☐	Nein ☐	Weiß nicht / Keine Angabe ☐

5.7 Hat die Corona-Pandemie insgesamt aus Ihrer Sicht Auswirkungen auf die <u>Gesundheit</u> Ihres Kindes / Ihrer Kinder?

Ja, negative Auswirkungen ☐	Ja, positive Auswirkungen ☐	Nein, keine Auswirkungen ☐	Weiß nicht / Keine Angabe ☐

Vielen Dank!
