# Supplementary material for: Quality of life and healthcare utilization during the COVID-19 pandemic are more restricted in chronically ill than in healthy children: a tertiary care children’s hospital experience
Source: Eur J Pediatr. 2024 Jan 23;183(4):1801–10. doi: 10.1007/s00431-023-05382-6 (PMC11001739; doi:10.1007/s00431-023-05382-6)
Supplement: Supplementary file 2 — Supplementary file2 (PDF 222 KB) [file 431_2023_5382_MOESM2_ESM.pdf]

Universitätsklinikum Tübingen

Questionnaire

Date:
No.:

Please fill in completely and only check one answer box at a time. If you have any questions, please contact one of the study physicians.

Time: ~ 10 minutes

Consent to Participation:

Thank you for agreeing to participate in the study mentioned above. Have you been adequately informed, have your questions been answered and do you wish to participate in the above survey study?

Yes ☐	No ☐

Part '1' – Participants

1. Are you parent or custodian accompanying the child/children today?

Parent ☐	Custodian ☐	Not applicable or no information ☐

1.1 Which age group do you belong to?

16-20 years ☐	21-30 years ☐	31-40 years ☐	41-50 years ☐	51-60 years ☐	61-70 years ☐	> 70 years ☐

1.2 What is your gender?

Male ☐	Female ☐	Divers ☐

1.3 What is your job?

Student ☐	Research associate ☐	Employee ☐	Self-employed person ☐	Unemployed ☐	Pensioners ☐	Not applicable or no information ☐

1.4 Do you work in the healthcare sector? (e.g. nursing, physician, medical assistant)

Yes ☐	No ☐	No information ☐

Part ‘2’ – New type of corona virus / corona pandemic

2.1 Have you or other persons living in the same household as your child/children been infected with COVID-19? (Attention: The children themselves are not meant!)

Yes, with positive test result ☐	Yes, without test result ☐	No, with negative test result ☐	No, without test ☐	I do not know / no information ☐

2.2 Do you know someone who has / has been infected with COVID-19 or do you work with people who have / have been infected with COVID-19?

Yes ☐	No ☐	I do not know / no information ☐

2.3 How worried are you about being infected with COVID-19 yourself?

Very high ☐	High ☐	Moderate ☐	Low ☐	Not at all ☐	I do not know / no information ☐

2.4 How concerned are you that your child(ren) will be diagnosed with COVID-19?

Very high ☐	High ☐	Moderate ☐	Low ☐	Not at all ☐	I do not know / no information ☐

The following questions 2.5 - 2.9 refer to the period of the entire corona pandemic!

2.5 How high do you think the risk is for your child(ren) to become infected during everyday errands (e.g. shopping at the supermarket, visiting a playground)?

Very high ☐	high ☐	Moderate ☐	Low ☐	Not at all ☐	I do not know / no information ☐

2.6 How high do you think the risk is for your child(ren) to become infected with Corona virus during a visit to your regular pediatrician/general practitioner?

Very high ☐	High ☐	Moderate ☐	Low ☐	Not at all ☐	I do not know / no information ☐

2.7 How high do you think the risk is for your child(ren) to become infected with Corona virus during a visit to a pediatric clinic?

Very high ☐	High ☐	Moderate ☐	Low ☐	Not at all ☐	I do not know / no information ☐

2.8 Has the Corona pandemic affected your child(ren)'s mental and/or physical health?

Yes, influenced in a negative way ☐	Yes, influenced in a positive way ☐	No, no influence ☐	I do not know / no information ☐

2.9 as the Corona pandemic affected your child(ren)'s everyday quality of life?

Yes, influenced in a negative way ☐	Yes, influenced in a positive way ☐	No, no influence ☐	I do not know / no information ☐

Part '3' – Child(ren)

3.1 How many children do you have?

1 child	2 children	3 children	4 children	> 4 children	No information
☐	☐	☐	☐	☐	☐

3.2 How old are your children? (multiple answers are possible here!)

Child	0-1 years (Meaning: Within the first 24 months of life)	2-5 years	6-12 years	13-18 years	>18 years	No information
#1	☐	☐	☐	☐	☐	☐
#2	☐	☐	☐	☐	☐	☐
#3	☐	☐	☐	☐	☐	☐
#4	☐	☐	☐	☐	☐	☐
#5	☐	☐	☐	☐	☐	☐
#6	☐	☐	☐	☐		☐
#7	☐	☐	☐	☐		☐

3.3 Does one of your children have a chronic disease (e.g. asthma, neurodermatitis, inflammatory bowel disease, rheumatism, etc., see definition)?

Yes ☐	No ☐
➔ Continue from section 4 (continue from page 5 to end)	➔ *Continue only from section 5 (from page 9 to end)*

Definition of chronic disease(s) (in childhood):

- llness/condition has existed for at least 3 months (note: children under 1 year not to be assessed here)[1]
- Illness/condition limits age-appropriate activities and/or has recurring need for medical care/check-ups[1]
- *In general, a diagnosis by a pediatrician for a period longer than 3 months is considered a chronic condition.*

[1]Schmidt, Silke & Thyen, Ute. (2008). Was sind chronisch kranke Kinder?. Bundesgesundheitsblatt - Gesundheitsforschung - Gesundheitsschutz. 51. 585-591. 10.1007/s00103-008-0534-5.

3.3.1 If 'YES', with which of your children do you present today with an appointment or acute illness?

My child(ren) with chronic disease ☐	My otherwise healty child(ren) ☐

Part '4' – Families with children suffering from chronic disease(s) / Procedure with children suffering from chronic disease(s)

4.1 How many of your children have a chronic health condition?

1 child	2 children	3 children	4 children	> 4 children	No information
☐	☐	☐	☐	☐	☐

4.2 What type of chronic disease does your child(ren) have? (multiple answers are possible here!)

Child	Airway (e.g., asthma)	Liver and/or intestine	Heart	Kidney	Rheumatism / joints	Hämatology / oncology (e.g., cancer)	Metabolic (e.g., diabetes)	Others	I do not know / no information
#1	☐	☐	☐	☐	☐	☐	☐	☐	☐
#2	☐	☐	☐	☐	☐	☐	☐	☐	☐
#3	☐	☐	☐	☐	☐	☐	☐	☐	☐
#4	☐	☐	☐	☐	☐	☐	☐	☐	☐
#5	☐	☐	☐	☐	☐	☐	☐	☐	☐
#6	☐	☐	☐	☐	☐	☐	☐	☐	☐
#7	☐	☐	☐	☐	☐	☐	☐	☐	☐

4.3 Was (or is) your chronically ill child/children affected by COVID-19?

Yes, with positive test result ☐	Yes, without test result ☐	No, with negative test result ☐	No, without test ☐	I do not know / no information ☐

4.4 Do any of your chronically ill children take medication on a regular/permanent basis due to their condition?

Yes ☐	No ☐	I do not know / no information ☐

4.4.1 If 'YES', did the Corona pandemic cause supply problems with needed medications?

Yes	No	I do not know / no information
☐	☐	☐

4.5 Does the chronic condition of one / several of your children necessitate regular outpatient doctor visits and / or inpatient hospital admissions?

Yes	No	I do not know / no information
☐	☐	☐

4.6 Have there been any rescheduling or cancellations of outpatient appointments with your pediatrician/general practitioner or at a specialty consultation (e.g., a pediatric clinic) for one of your chronically ill children due to the Corona pandemic?

Yes, the clinic/doctor's office canceled the appointment ☐	Yes, I/we have cancelled the appointment ☐	No, no rescheduling ☐	No, no doctor's visit was planned / required ☐	I do not know / no information ☐

4.6.1 If 'YES': Where was this appointment scheduled?

Pediatrician / general practitioner ☐	Special consultation (e.g. asthma consultation in a pediatric clinic) ☐	Outpatient intervention / diagnostics (e.g., outpatient surgery, imaging such as X-ray/CT/MRI, etc.) ☐	Other (e.g. physiotherapy, rehab, etc.) ☐	I do not know / no information ☐

4.6.2 If 'YES', were necessary appointments for regular preventive examinations (U-examinations) or vaccinations affected by this?

Yes ☐	No ☐	I do not know / no information ☐

4.6.3 If 'YES, I/we cancelled the appointment': did you cancel or reschedule the outpatient appointment because of concerns about contracting Corona virus?

Yes ☐	No ☐	I do not know / no information ☐

4.6.4 If 'YES, the clinic/practice canceled the appointment,' were alternative options offered as a substitute for presenting at the specialty consultation, such as video consultation, telephone consultation, extensive telephone calls, or similar?

Yes ☐	No ☐	I do not know / no information ☐

4.7 Have you taken advantage of non-contact medical care offerings such as video consultations, telephone consultations, extensive telephone calls, or equivalent?

Yes ☐	No ☐	I do not know / no information ☐

4.7.1 If 'YES', how well could this replace the face-to-face presentation at the specialty consultation?

Very good ☐	Good ☐	Moderate ☐	Low ☐	Not at all ☐	I do not know / no information ☐

4.8 In the context of the Corona pandemic, would you like to see more offerings of non-contact medical care, such as video consultations, telephone consultations, extensive telephone calls, or equivalent?

Yes ☐	No ☐	I do not know / no information ☐

4.9 Have there been any rescheduled or cancelled inpatient appointments for any of your chronically ill children due to the Corona pandemic?

Yes, the clinic/doctor's office canceled the appointment ☐	Yes, I/we have cancelled the appointment ☐	No, no rescheduling ☐	No, there were no inpatient appointments scheduled ☐	I do not know / no information ☐

4.9.1 If ‘YES, I/we cancelled the appointment’, have you cancelled or rescheduled inpatient appointments due to concerns about contracting Corona virus?

Yes ☐	No ☐	I do not know / no information ☐

4.10 If there were any cancellations or rescheduling of outpatient and/or inpatient appointments: *In your opinion*, how much did this negatively affect your child's health?

Very much ☐	Much ☐	Moderate ☐	Low ☐	Not at all ☐	I do not know / no information ☐	Not applicable / No appointment cancellations ☐

4.11 Have the treating physicians / therapists provided you with information on managing corona virus with your chronically ill child / children?

Yes, plenty of information ☐	Yes, but too less information ☐	No, no information ☐	I do not know / no information ☐

4.11.1 Did / Would you have (had) more need for information (e.g., accessible online) on managing corona virus in your chronically ill child/children?

Yes ☐	No ☐	I do not know / no information ☐

4.12 From your perspective, has the Corona pandemic as a whole had an impact on the health of your child/children with chronic illness?

Yes, negative impact ☐	Yes, positive impact ☐	No, no impact ☐	I do not know / no information ☐

4.13 In the event of an acute illness in one of your children, would you avoid or delay a doctor's visit during the Corona pandemic *in general*?

Yes ☐	No ☐	I do not know / no information ☐

4.14 In the event of an acute illness but *minor problem in your opinion* *(e.g., cough, diarrhea)* in one of your children, would you avoid or delay a visit to the doctor during the Corona pandemic?

Yes ☐	No☐	I do not know / no information ☐

4.15 In the event of an acute illness in one of your children that *may be life-threatening in your opinion*, would you avoid or delay a doctor's visit during the Corona pandemic?

Yes ☐	No ☐	I do not know / no information ☐

Part ‘5’– Families with otherwise healthy children / Procedure for healthy children

PLEASE NOTE:

If you have only chronically ill children / a chronically ill child please DO NOT fill in!

5.1 Was (or is) your otherwise healthy child/children suffering from COVID-19?

Yes, with positive test result ☐	Yes, without test result ☐	No, with negative test result ☐	No, without test ☐	I do not know / no information ☐

5.2 Have there been any rescheduled or cancelled appointments with your pediatrician/general practitioner for any of your children due to the Corona pandemic?

Yes, the clinic/doctor's office canceled the appointment ☐	Yes, I/we have cancelled the appointment ☐	No, no rescheduling ☐	No, no doctor's visit was planned / required ☐	I do not know / no information ☐

5.2.1 If ‘YES’, what type of medical presentation was scheduled?

Regular preventive medical check-ups (e.g. U-examinations, vaccinations, etc.) ☐	Acute presentation (e.g. respiratory infection, abdominal pain, etc.) ☐	Other (e.g., picking up a prescription, being excused for school classes) ☐	No information ☐

5.2.2 If ‚YES, I/we cancelled the appointment’, did you reschedule or cancel one or more appointments due to concerns about contracting Corona virus?

Yes ☐	No ☐	I do not know / no information ☐

5.2.3 If ‘YES’, how much do you think the appointment rescheduling or cancellation negatively affected your child's health?

Very much ☐	Much ☐	Moderate ☐	Low ☐	Not at all ☐	I do not know / no information ☐

5.3 Should any of your children be hospitalized during the Corona pandemic?

Yes ☐	No ☐	I do not know / no information ☐

5.3.1 If ‘YES’, did you decline your child's inpatient admission at your own request because of concerns about COVID-19, or did you leave the hospital earlier than planned at your own request?

Yes ☐	No ☐	I do not know / no information ☐

5.3.1.1 If 'YES', how much do you think this has negatively affected your child's health?

Very much ☐	Much ☐	Moderate ☐	Low ☐	Not at all ☐	I do not know / no information ☐

5.4 In the event of an acute illness in one of your children, would you avoid or delay a doctor's visit during the Corona pandemic *in general*?

Yes ☐	No ☐	I do not know / no information ☐

5.5 In the event of an acute illness but *minor problem in your opinion* *(e.g., cough, diarrhea)* in one of your children, would you avoid or delay a visit to the doctor during the Corona pandemic?

Yes ☐	No ☐	I do not know / no information ☐

5.6 In the event of an acute illness in one of your children that *may be life-threatening in your opinion*, would you avoid or delay a doctor's visit during the Corona pandemic?

Yes ☐	No ☐	I do not know / no information ☐

4.16 From your perspective, has the Corona pandemic as a whole had an impact on the health of your child/children with chronic illness?

Yes, negative impact ☐	Yes, positive impact ☐	No, no impact ☐	I do not know / no information ☐

Thank you!
